# The Role of Indian Caste Identity and Caste Inconsistent Norms on Status Representation

**DOI:** 10.3389/fpsyg.2017.00487

**Published:** 2017-03-31

**Authors:** Sindhuja Sankaran, Maciek Sekerdej, Ulrich von Hecker

**Affiliations:** ^1^Department of Psychology, University of WarsawWarsaw, Poland; ^2^Institute of Psychology, Jagiellonian UniversityKraków, Poland; ^3^School of Psychology, Cardiff UniversityCardiff, UK

**Keywords:** black sheep effect, caste identity, norm-violation, social identity threat, status

## Abstract

The Indian caste system is a complex social structure wherein social roles like one’s profession became ‘hereditary,’ resulting in restricted social mobility and fixed status hierarchies. Furthermore, we argue that the inherent property of caste heightens group identification with one’s caste. Highly identified group members would protect the identity of the group in situations when group norms are violated. In this paper, we were interested in examining the consequence of caste norm violation and how an individual’s status is mentally represented. High caste norms are associated with moral values while the lower caste norms are associated with immorality. We predicted a ‘black sheep effect,’ that is, when high caste individuals’ group identity (caste norm violation condition) is threatened their salient high caste identity would increase, thereby resulting in devaluing the status of their fellow in-group member if the latter is perceived as perpetrator. We presented participants with a social conflict situation of a victim and a perpetrator that is ‘*Caste norm consistent’* (Lower caste individual as a perpetrator and higher caste individual as a victim) and vice versa *‘Caste norm inconsistent’* condition (higher caste individual as perpetrator and lower caste individual as a victim). Then, participants had to choose from nine pictorial depictions representing the protagonists in the story on a vertical line, with varying degrees of status distance. Results showed evidence for the black sheep effect and, furthermore, revealed that no other identity (religious, national, and regional) resulted in devaluing the status of fellow in-group member. These results help us understand the ‘black sheep’ effect in the context of moral norms and status representation and are discussed in the framework of the Indian society.

## Introduction

People in general belong to many social categories that could either be achieved, such as one’s profession, or inherited, such as one’s gender. The consequences of social categorizations are often not only seen in the dynamics of social interactions, but also in the way social status is represented. For the present research, the Indian/Hindu caste system is of interest, which is an integral feature of the Indian societal structure. The caste system provides a hierarchy of social roles that hold inherent characteristics and, more importantly, remain stable throughout life (Dirks, 1989). An implicit status is attached to one’s caste which historically changed from the social roles to hereditary roles. This, created status hierarchies on hereditary basis with limited social mobility. For instance, individuals born into the highest caste, that is, the *Brahmin* caste have usually been priests and scholars. Individuals born into the *Kshatriya* caste have been warriors and kings. Individuals born into the *Vaishya* caste have been merchants. Finally, individuals born into the *Shudra* caste have been laborers. Besides, there was an additional ‘out-casted’ group called the *Dalits* or the ‘untouchables’ who occupied the lowest step of the social ladder (see Ambedkar, 1925/1989; Pick and Dayaram, 2006). In modern India, the Indian government introduced a categorization scheme in which the untouchable castes were categorized as scheduled castes (SC), the backward tribes were categorized as scheduled tribes (ST) and the disadvantaged castes as other backward castes (OBC). The Forward caste (FC) community generally constitute the high caste group. The SC, ST, and OBC comprising the historically disadvantaged groups, were provided job opportunities by the government through affirmative action (Sheth, 1987; Kumar, 2001; Gupta, 2005; Dreze and Khera, 2009). The FC has historically been and, continues to be, in a strong socioeconomic position with the highest status in society^1^. Thus, one of the main objectives of the present research was to examine how status is cognitively represented in the Indian society as a consequence of the way caste is perceived^2^. Even now, people in India continue to define their self-identity by means of the caste they belong to and the social group that they find themselves in. Caste membership is thus ingrained in the society and there is considerable reason to claim that caste as a type of social identity would probably be one of the most salient identities in the Indian context. This aspect is addressed by Social Identity Theory (Tajfel and Turner, 1986), to which we now turn.

### Social Identity as a Basis for Caste Identity

Social identity claims that people derive an important part of their identity from an affirmation of membership with the group they belong to. Tajfel and Turner (1986) suggested that any group (e.g., social class, family, football team etc.) can act as a source of pride and self-esteem, therefore, we tend to enhance our self-esteem by promoting and endorsing the *status* of the group we belong to, the so-called “in-group” (as opposed to “out-groups” being those groups that we do *not* belong to, see also Hogg and Turner, 1987). The Indian societal structure provides a fertile ground to examine the interactive roles of multiple identities like religious, national, regional (north vs. south), class and caste wherein one could discard or fuse these identities for the benefit of societal functioning (Miller et al., 1990; Miller and Bersoff, 1992). But many researchers have stressed the importance and the influence of caste as an integral social identity among many South Asians compared to other social identities like gender and ethnicity (for example, Gayer, 2000; Mand, 2006). It has in fact been argued that caste identity may override other social identities, because of its primary importance for many South Asians (Judge and Bal, 2008). We argue that in the context of status representation, caste identity (as opposed to religion, national and regional identities) would be the most prominent identity in explaining the differences in status perception, due to the inherent associations of caste and status. Thus, according to social identity theory, individuals would strive to maintain a positive image of their caste identity. We further argue below that caste identity will especially be more salient for high caste individuals.

A strong caste identity could provide feelings of belongingness or self-esteem, thereby relying on some caste norms. Particularly, it is known that high caste individuals see caste identity as a more stable construct wherein this identity is inherited at birth. They tend to essentialise their identity and this is predominantly attributed to the feelings of connectedness with previous generations of one’s caste group. High caste individuals also develop feelings of temporal continuity, positive distinctiveness, and heightened self-esteem from essentialisation of their caste identity (Jaspal, 2011). In fact, in a study conducted by Cotterill et al. (2014), it was argued that the caste system tends to be legitimized through the ideology of Karmic beliefs (beliefs that general good or bad deeds in one’s life are rewarded or reprimanded by being born into a high or low caste in the next life) especially by those high on social dominance orientation (SDO), that is, those who demonstrate a general preference for hierarchical social relations (Sidanius and Pratto, 1999; Pratto et al., 2000). Furthermore, when members of higher castes essentialise their caste identity (Mahalingam, 2003) they permit themselves to stigmatize members of the lower castes. The low caste members or the *Dalits* on the other hand, do not believe that their caste identity is inherited and therefore do not essentialise it. They may thus enhance their self-efficacy, through the possibility of social mobility, based on the idea that caste identity can be seen as less permanent (Mahalingam, 2003). We thus argue that caste identity is more salient amongst high caste individuals due to the belief they have about being privileged to have inherited this positive image of high caste at birth. Low caste individuals would not have a salient caste identity because they believe that this identity is not essentialised and belonging to this group has negative consequences.

### Social Identity Threat and Caste Norms

Social identity effects are based on the protection of self-concepts (Tajfel and Turner, 1986) and thus any threat to this self-concept would be associated with strong identity effects. Research has shown that highly identified group members would find ways to protect their in-group identity (see Spears et al., 1997). However, Branscombe et al. (1999) claim that threat to one’s social identity in fact depends on the degree of group identification. For instance, they suggest that those who are highly identified with their in-group are more likely to show defensive responses than those who are not so highly identified. We can assume that high caste individuals who legitimize their inherent high caste would also show strong high caste identity.

So, what specifically could elicit an identity threat related to caste? We claim that norms and expectations that are associated with caste membership, when questioned, could fundamentally be a source of threat. In fact, it is most commonly seen that a person engaging in any sort of norm violation (especially of the higher caste) is ostracized and devalued (Mahalingam, 2007). One of the most deeply rooted caste norms relates to marriage. For instance, when people violate the norm of marrying within one’s own caste by engaging in inter-caste marriage, the higher caste individual is believed to bring shame to the family and this norm transgression is considered to be immoral. Branscombe et al. (1999) argued that when an identity related to the morality value is threatened, high identifiers will show more defensive reactions. We therefore argue that the threat to one’s own caste, if related to moral values or norms would motivate strong caste identifiers to alleviate this threat and protect their identity.

For many years, the high caste members in general had greater status in the society, and viewed themselves as living to higher moral standards and values, as compared to low caste individuals (Mahalingam, 2003). It is generally believed that high caste individuals hold qualities related to wisdom, intelligence, honesty, austerity, and morality while low caste individuals possess qualities of dullness, stupidity, immorality, impurity, and other negative qualities (Deshpande, 2010). These ancient established norms carried over into modern day Indian society and thus certain norms were explicitly attached to a caste type. Thus we can argue that morality is perhaps a significant value attached to one’s caste and violating such a norm could be a source of threat particularly amongst high identifiers. As introduced earlier, inter-caste marriages can be seen as a typical norm-violation in India and are often viewed as ‘polluting’ the sanctity of the caste system (Dube, 2001), thereby touching upon the value of morality. Marriages between high and low caste persons are especially harshly punished and sometimes lead to public lynching of couples or their relatives, murder (of the bride, groom or their relatives), rape, public beatings and other sanctions (NYU 2007, p. 11 as cited in Hoff et al., 2009). In fact, in Northern India, inter-caste marriages frequently result in family members choosing to kill the couple (Flintoff, 2010; Goli et al., 2013). Thus, when a high caste member commits norm violation s/he is devalued in society. This effect can especially be understood by the ‘Black sheep effect’ (BSE) wherein people in general derogate deviant in-group members (Marques and Paez, 1994).

### Norm Violation Effects and Identity

When a norm is violated, members often perceive this deviant behavior as potentially threatening to the group identity, and therefore deal with the deviance in order to reduce the threat (Jetten and Hornsey, 2014). However, research has shown that the tendency for a group to defend the threat depends on the extent to which an individual is identified with the group (Marques and Paez, 1994). Those who are not as much identified with the group, are typically less motivated to protect one’s social identity (Spears et al., 1997; Rimal and Real, 2005). It can thus be understood that high identifiers would show greater motivation to engage in in-group protection to defend the threat (Doosje et al., 1995). We argue that high caste individuals would be high identifiers with their caste, and low caste individuals would be low identifiers with their caste. However, we claim that in-group identity protection will be seen in the form of black sheep effect and not as in-group favoritism. In certain situations, in-group members are known to exclude undesirable members from the in-group in order to maintain a positive and distinctive social identity (Marques and Paez, 1994). For instance, research by Otten (2009) claims that an aggressive social interaction between a victim and a perpetrator would lead to generally biased responses that could either lead to in-group favoritism or black sheep effect; the latter effect being most likely to occur in situations. More specifically it is said that in-group favoritism is particularly observed when the deviant behavior of the perpetrator was ambiguous or unintentional (Duncan, 1976; Sagar and Schofield, 1980). However, when there is explicit evidence suggesting that in-group perpetrators deliberately “committed the crime” (see also Abrams et al., 2000; Marques et al., 2001) one would observe the black sheep effect. Wang et al. (2016) also found neural evidence showing that intentional aggressive interactions result in patterns of the black sheep effect. Thus, there is some evidence indicating that aggressive, intentional, and unambiguous interactions would lead to more in-group derogations.

Furthermore, this pattern of in-group derogation tends to be more distinct among individuals who are highly identified with their group (Biernat et al., 1999) than those who are not. High-caste individuals would indeed be high identifiers, owing to the notion of ‘being born into’ one’s high caste and thus would be especially motivated to protect one’s in-group by excluding the undesirable member (see Branscombe et al., 1999). Furthermore, Castano et al. (2002) explain that well- established group members (high caste members) are especially aware of the pertaining rules and norms and therefore, any kind of deviance from such norms would pose a threat to one’s group identity, which will be responded to by devaluating the perpetrating in-group member and seeing him/her as low in typicality. Research by Stamkou et al. (2016) further also add that especially those who highly identify with one’s in-group perceive the in-group deviant as less typical of the in-group. Thus, in general, norm-deviating in-group members are seen as more negative than non-deviating members (Marques et al., 1998, 2001; Pinto et al., 2010). Also, according to the threat classification by Branscombe et al. (1999), it is seen that in the face of moral value threat, high identifiers (high caste individuals) are most likely to respond to such a threat by engaging in defensive reactions (black sheep effect) and wanting to be different from the deviant member of the group. We would thus argue that high caste individuals who are also high identifiers with their caste would devalue another in-group member committing norm transgression (that is aggressive and intentional) and would find the transgression morally unacceptable in order to protect their threatened social identity.

Elaborating on the black sheep effect, according to subjective group dynamics theory (SGD; Marques et al., 1998; Abrams et al., 2000) group members are motivated to maintain a positive social identity. This motivation then results in positive evaluations of in-group conformers and negative evaluations of in-group deviants (Marques et al., 1998, 2001). In a similar vein, Stamkou et al. (2016) found that higher ranked group members showed more preference for norm followers than norm violators. They suggest that this could be because higher ranked members were more threatened by the norm violator’s challenge to the status quo. Thus we can argue that high caste individuals would be more motivated to protect one’s in-group identity by making negative evaluations of the deviant member. Likewise, according to relational models theory (RMT, Fiske, 1991, 1992), a derogation of in-group member in order to protect a group identity and integrity is explained by a transgression of moral norms regulated by specific in-group relations. In our context it particularly refers to moral motives for unity and hierarchy. Unity is aimed at caring for and supporting the integrity of in-group by avoiding or eliminating threats of contamination. When a group member commits a moral violation, the whole group feels contaminated and shamed until it purifies itself. Hierarchy in turn is aimed at maintaining linear orderings of social status where subordinates are motivated to respect and obey, and superiors to guide, protect, but also take moral responsibility for the actions of their subordinates (for review of all moral motives see Rai and Fiske, 2011). Thus, high caste individuals who break the strongly ingrained high caste norm of morality, purity, self-control, and pastoral care must expect group aversion or even a punishment. We were therefore interested in identity threat in the form of caste norm violation, and the ensuing cognitive representations of caste and status, which could be identity-maintaining. We assume in this context only the caste-based identity will be activated whilst other identities, such as religion, national and regional affiliation, will not play a role.

### Caste and Social Consequences

One of the most common social problems of the caste system was the discrimination of low caste members as explained earlier. In 1950, independent India’s constitution banned caste-based discrimination and in order to compensate for historical injustices the authorities introduced *quotas* in government jobs and educational institutions to improve the quality of life of low castes (“What is India’s caste system?”, 2016, February 26., para. 13). A *reservation system* was introduced wherein a certain number of seats were reserved for members of the lower castes at places of higher education and government jobs. However, this legislation was soon met with a lot of resistance from the high caste community who felt that the system was not meritocratic, and provided an unjust advantage to the low caste members (e.g., Siddique, 2011). We believe that the reservation system is one of the most important social consequences of the caste system in modern times, and attitudes toward the system would have to be a reflection of one’s caste identity.

### The Present Research

It was argued that social identity would play an integral role in the way people represent status. Threat to one’s social (caste identity) in the form of norm violations would thus result in engaging in in-group protection. It is important to note that we refer to norm violations as behavior of fellow in-group members and not to one’s own norm violations. Therefore, the aim of this study was to examine the role of caste-norm inconsistent (violating) vs. caste norm-consistent (non-violating) situations in making mental representations of status. Furthermore, we also wanted to examine how high caste and low caste individuals make moral judgments in situations of norm violation vs. non-violation. We predict that caste identity threat would be most salient for high caste individuals because these individuals in general have higher levels of caste identity. In general, we predicted that high caste individuals would show a black sheep effect when caste norm violations are introduced. We thus expected that in the face of identity threat, in a norm-inconsistent condition, there will be salient caste identity resulting in a mental representation that reflects the devaluation of an in-group perpetrator (black sheep effect), and lowered moral acceptability of an in-group perpetrator’s action, particularly amongst high caste individuals. Moreover, we expect that this black sheep effect is mediated by high caste identity (and not mediated by regional, religious or national identity).

In this study, we used vignettes describing a social conflict situation which comprised norms of violence and morality, as reflecting common norms in the Indian context. The social conflict situation always entailed an intentional aggressive interaction between a victim and a perpetrator. Participants read two stories that depicted two protagonists as either a victim or perpetrator. The protagonists were either of high or low caste which was depicted using implicit stimuli of names and faces (pre-tested). They then had to choose and pick, as a response, one out of nine pictorial depictions that represented the protagonists in the story on a vertical line with varying degrees of status distance. At the end of each story participants also had to rate the moral acceptability of the behavior that took place between the protagonist and the victim. This procedure was chosen based on the general idea that status judgments are supported by an automatic simulation of vertical location (von Hecker et al., 2013; see also Schubert, 2005 for the same argument on social power). We assume that abstract concepts such as status can be mentally represented in an embodied way (Niedenthal et al., 2005). Giessner and Schubert (2007) showed that information about a leader’s power influenced participants’ vertical positioning of the leader. The paradigm used here involves such status representations via the vertical dimension. In this study, we interpret black sheep effect in the form of status evaluations wherein the in-group member is not necessarily excluded but devalued in status instead. That is, when the perpetrator is portrayed as someone from their in-group (high caste), they would then derogate that individual by representing him as having lower status. Low caste individuals on the other hand would not differ in the way they represent status regardless of the condition, that is, they would generally show the trend of depicting a perpetrator as having greater status than a victim regardless of whether the perpetrator is of high or low caste^3^. Finally, we also predict that high caste identity will also mediate the relationship between caste affiliation and social outcomes of caste like endorsement of affirmative action. Thus, high caste individuals’ opposing attitude toward educational affirmative action will be mediated by high caste identity.

## Materials and Methods

### Participants

One hundred and two South Indians from various parts of Chennai, India, were recruited in person by choosing a convenience sampling technique. The participants were approached from various parts of the city and explained the study with examples and in the local language – Tamil. As a cover story, they were informed that the survey was part of a large research project that would help in understanding the Indian society better. The test materials were administered using paper and pencil. The researcher stayed in the area until the participants had completed the survey and they answered questions if necessary. Participants volunteered to participate for free in this research (age *M* = 33.25, *SD* = 12.46; 56 female; 45 high caste). Descriptive details of participant demographics are outlined in **Table 1**.

**Table 1:** Frequencies and percentage for all demographic variables.

	*N*	Percentage
**Gender**		
Male	46	45.1
Female	56	54.9
**Religion**		
Hindu	62	60.8
Muslim	18	17.6
Christian	22	21.6
**Perceived SES**		
Low	18	17.7
Middle	72	70.6
Upper	12	11.7
**Education**		
Middle School	30	29.4
School	28	27.5
Graduate	44	43.2
**Caste**		
High Caste	45	44.2
Low Caste	57	56.5

#### Design and Procedure

All participants completed the demographic questions and were then instructed that they would be reading a few stories and were required to answer a few questions based on them. Participants were then randomly assigned to one of the two conditions. (i) caste-norm consistent, wherein the perpetrator was of a low caste and the victim was of high caste, and (ii) caste-norm inconsistent, wherein the perpetrator was of high caste and the victim was of low caste. Participants then read the stories pertaining to each condition and answered questions on status representation and moral acceptability for each story. This was then followed by answering questions on attitudes toward affirmative action, about their national, regional, caste and religious identities and, finally, the manipulation check. At the end of the questionnaire all participants were debriefed.

### Materials and Measures

#### Caste Stimuli Materials

Names and faces were pre-tested, from which four names and faces emerged; two belonging to low caste and two belonging to high caste from South India. The names contained the first name and a surname, indicative of a certain caste. The length of the name was not controlled since certain surnames are more indicative of the caste than others. The low caste names included Selvaraj Mani and Kannan Pandian. The high caste names included Murali Raman and Ravi Krishnamurthy. The characteristic features used to distinguish faces belonging to different caste included (i) Skin color, wherein a high caste face typically had lighter skin color and a low caste face had a darker skin color (ii) Facial hair, wherein a higher caste face would have less to no facial hair while a lower caste face would have more facial hair, and (iii) General grooming, wherein a high caste face looked more groomed than a low caste face. Participants were never explicitly informed that the people in stories were either of high or low caste.

#### Social Conflict Stories

Participants read two stories that described a social threat situation wherein two protagonists in a story were either a victim or a perpetrator. The particular conflict situations, involving issues of violence and morality, were chosen because of their commonness in the Indian society and because such issues are heavily rooted in distinguishing high and low caste characteristics. In the caste norm-consistent condition all participants read two stories in a South Indian context, wherein the victim was portrayed as a high caste member and the perpetrator was portrayed as a low caste member, based on the caste associations which names and faces would elicit. For example, “At the junction of Mount Road there was a huge traffic jam where two cars collided. While nobody got hurt, the drivers jumped out of their cars and started hurling verbal abuses at each other. That quarrel soon turned into a proper fight when *Selvaraj Mani* jumped out from his car and attacked *Murali Raman*. Murali Raman was pulled out of his car and was punched hard by *Selvaraj Mani*. *Murali Raman* finally fell to the ground and shouted apologies, but *Selvaraj Mani* did not stop kicking him until the police came and pulled the two drivers apart.” In the caste norm-inconsistent condition, participants also read two South Indian stories wherein the victim was portrayed as a low caste member and the perpetrator was portrayed as a high caste member. For example, “At the junction of Mount Road there was a huge traffic jam where two cars collided. While nobody got hurt, the drivers jumped out of their cars and started hurling verbal abuses at each other. That quarrel soon turned into a proper fight when *Murali Raman* jumped out from his car and attacked *Selvaraj Mani*. *Selvaraj Mani* was pulled out of his car and was punched hard by *Murali Raman*. *Selvaraj Mani* finally fell to the ground and shouted apologies, but *Murali Raman* did not stop kicking him until the police came and pulled the two drivers apart.” At the end of each story, two photos were presented of the faces and their respective names below the photos. Thus, the low caste name was presented below the low caste photo and the high caste name was presented below the high caste photo. Two different conflict situations were chosen. One was about a traffic jam situation and the second was about a situation in the office where two colleagues get into a dispute (see Appendix 1 for all stories). The stories were counterbalanced within the South Indian context and also between conditions. All stories were in Tamil^4^.

#### Demographic Questions

Participants completed a survey that included several demographic questions indicating one’s religion, perceived socio-economic status, education and caste. The caste categories included most backward caste (MBC), backward class (BC), ST, SC, and FC. Participants were asked to indicate their caste by choosing one of the categories. For the purpose of the research, MBC, BC, ST, and SC were grouped together as *low caste* and FC remained as *high caste*. **Table 1** shows all frequencies and percentages for other demographic variables.

#### Status Representation

**FIGURE 1:**
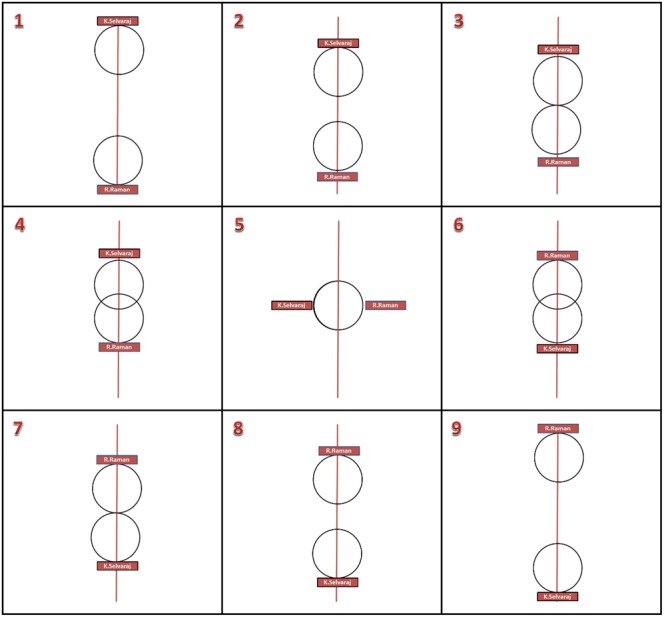
**Sample status distance dependent variable**.

After reading the stories participants had to choose one out of nine pictorial representations which, according to them, best depicted the social status between the two protagonists in the story. Each picture comprised a vertical line with two circles representing each of the protagonists with varying degrees of status. A wider distance between the two circles on the vertical dimension indicated a greater status difference (see **Figure 1** for details). The first and the ninth picture represented greatest status distance but in different directions, for instance, the victim on the lower part of the vertical line and the perpetrator on the upper part of the vertical line, or the positions of victim and perpetrator reversed. Picture 5 had two circles merged into one on a vertical line, indicating equal status. These meanings were explained to participants. Distance was scored based on the picture whereby the picture representing the greatest distance got a score of 4 and the picture representing least distance got a score of 1. The picture representing equal status got a score of 0. Positive distance values were scored for congruent representation of status and stories, that is, victim at lower position and perpetrator at higher position. Negative distance values were assigned for incongruent representation of status and stories, that is, victim at higher position and perpetrator at lower position. Each participant received a score for each of the stories. All chosen distances pertaining to the South Indian stories were combined together, to yield a distance score.

#### Moral Acceptability

After choosing status representations for all stories, participants were asked to indicate on a scale (1 = *extremely unacceptable* to 5 = *extremely acceptable*) to what extent they perceived the behavior between the protagonist and the victim in the scenarios as morally acceptable.

#### Attitudes toward Reservation System

Participants rated a question on whether they supported or opposed the affirmative action policy (Indian quota system) on a scale (1 = *strongly oppose* to 5 = *strongly support*).

#### Multiple Identities

Participants were asked to indicate to what extent they identified themselves with being Indian, south Indian, high caste, low caste, and being religious on a scale (1 = *strongly do not identify* to 5 = *strongly identify*). All items were answered in Tamil.

#### Manipulation Check

At the end of the study participants were presented with all eight names of the protagonists and were asked to indicate which caste they belonged to by choosing high caste, low caste, or an unsure option.

## Results

**FIGURE 2:**
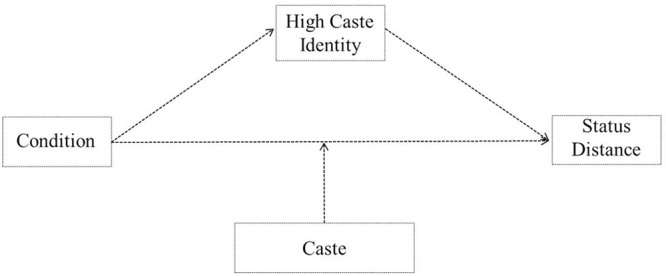
**Theoretical model of the relationship between condition (norm consistent vs. inconsistent), high caste identity and status distance as moderated by caste (high vs. low)**.

**FIGURE 3:**
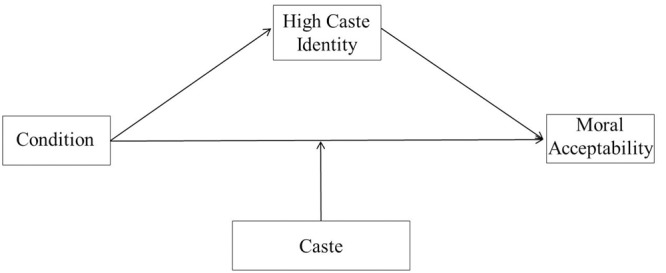
**Status representation as a function of caste (high vs. low) and condition (norm consistent vs. inconsistent)**.

Descriptive statistics of the manipulation check results are presented in **Table 2** which shows the response category frequencies for each of the protagonists. In general, the majority of participants categorized both South Indian high caste protagonists as belonging to the high caste and South Indian low caste protagonists as belonging to the low caste. Thus, we can assume that the names and faces of the protagonists were sufficient to identify the caste. Descriptive statistics and bivariate correlations of identity variables are presented in **Table 3**. For the purpose of the current study the status distances from the South Indian story 1 (*M* = 0.92, *SD* = 2.23) and South Indian story 2 (*M* = 0.61, *SD* = 2.49) were averaged and used since the sample was a South Indian sample. All participants read the stories and made individual status depiction for each story. To test our predictions, we checked whether caste-norm consistency (coded 0 as cast norm-inconsistent, 1 as caste norm-consistent) was associated with high-caste identity (mediator). In turn, high-caste identity was expected to predict status representation (DV) and moral acceptability (DV), but only amongst high caste members as compared to low caste members (moderator), as depicted in the theoretical model in **Figures 2, 3**, respectively. That is, we predicted a caste norm inconsistent condition would lead to incongruent status representations (victim seen as of higher status and perpetrator seen as of lower status), as mediated by high-caste identity, but that this effect might be seen only amongst high caste individuals. Similarly, we predicted that a caste-norm inconsistent condition would lead to less moral acceptability of the high-caste perpetrator, as mediated by greater high caste identity, and again only amongst high-caste individuals. We used model 5 in Process macro for SPSS (Hayes, 2013) to test these moderated mediation effects. In each case 20,000 bootstrap samples were used, 95% bias-corrected confidence intervals and unstandardized coefficients are reported. Religion, education, gender, perceived socioeconomic class, national, regional, and religious identities were all controlled for. Furthermore, since we wanted to examine the role of one identity in particular, it was essential to control for these other identities.

**Table 2:** Frequencies and percentage for caste categorized for each protagonist.

	High N(Percentage)	Low N(Percentage)	Unsure N(Percentage)
South Indian 1 (High)	77 (75.5)	9 (8.8)	16 (15.7)
South Indian 1 (Low)	9 (7.8)	83 (81.4)	11 (10.8)
South Indian 2 (High)	79 (77.5)	11 (10.8)	12 (11.8)
South Indian 2 (Low)	11 (10.8)	81 (79.4)	10 (9.8)

**Table 3:** Intercorrelations, means, and standard deviations for all identity variables and actual caste.

	Caste category	High caste identity	Low caste identity	National identity	South Indian identity	Religious identity
(1) Caste category	–	0.45^∗∗^	–0.58^∗∗^	0.03	–0.23ˆ*	0.15
(2) High caste		–	–0.37^∗∗^	–0.00	0.07	0.05
(3) Low caste			–	–0.02	0.18	–0.08
(4) National				–	–0.13	0.22ˆ*
(5) South Indian					–	0.06
(6) Religious						–
Mean/SD	0.35 (0.48)	2.75 (1.23)	2.97 (1.27)	3.78 (1.17)	3.40 (0.95)	3.46 (1.48)

**FIGURE 4:**
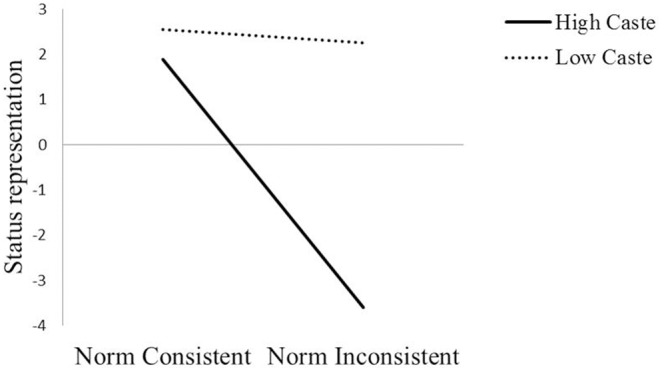
**Theoretical model of the relationship between condition (norm consistent vs. inconsistent), high caste identity and moral acceptability as moderated by caste (high vs. low)**.

In line with our predictions, we found that caste norm condition was related to status representation through the indirect effect of high-caste identity (*IE* = 0.24, 95% CI [0.06; 0.54]). The results showed that caste norm condition negatively predicted high-caste identity (*b* = -0.68, *t* = -2.81, *p* = 0.006, 95% CI [-1.17, -0.20]) and high-caste identity negatively predicted status distance (*b* = -0.35, *t* = -2.98, *p* = 0.004, 95% CI [-0.58, -0.12]). Thus, we see that caste norm inconsistency leads to higher caste identity which in turn results in incongruent status distances, however, only amongst high caste individuals. The conditional direct effect of caste norm condition on status representation was significant only among high-caste individuals (*b* = 4.84, *t* = 11.47, *p* < 0.0001, 95% CI [4.00, 5.68]) but not among low-caste individuals, (*b* = 0.39, *t* = 1.37, *p* = 0.175, 95% CI [-0.17, 0.95]). The interaction between condition and caste on status representation is graphically presented in **Figure 4**. The interaction between condition and caste is significant (*b* = 4.46, *t* = 8.52, *p* < 0.0001, 95% CI [3.42, 5.49]) wherein there is a significant difference between caste norm consistent and inconsistent condition amongst high-caste individuals (*b* = 0.37, *t* = 14.97, *p* < 0.0001, 95% CI [4.79, 6.26]) but no significant difference amongst low-caste individuals (*b* = 0.32, *t* = 1.10, *p* = 0.273, 95% CI [-0.26, 0.91]). In addition, results showed high caste individuals showed more status incongruent representations compared to low caste individuals in the norm inconsistent condition (*b* = -5.83, *t* = -17.87, *p* < 0.0001, 95% CI [-6.47, -5.17]). The analyses were repeated for each of the other identities (religious, national, regional, and low caste) that were used as mediators while controlling for the other. The indirect effects for lower caste identity (*IE* = -0.01, 95% CI [-0.09; 0.07]), South Indian identity (*IE* = -0.00, 95% CI [-0.07; 0.04]), national identity (*IE* = 0.00, 95% CI [-0.03; 0.08]) and religious identity (*IE* = -0.02, 95% CI [-0.16; 0.06]) were not significant.

**FIGURE 5:**
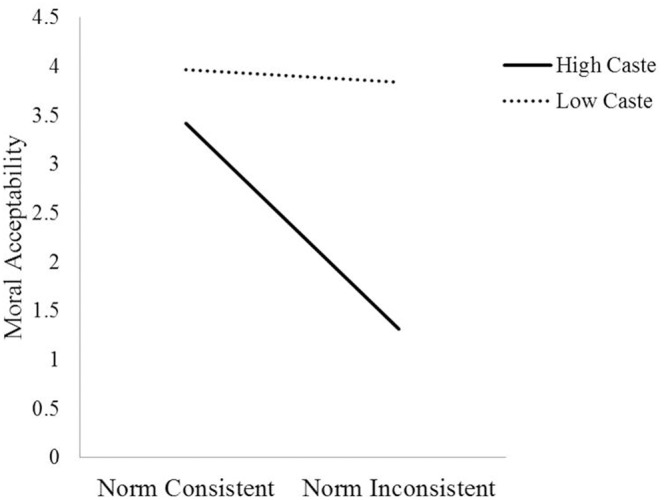
**Moral acceptability as a function of caste (high vs. low) and condition (norm consistent vs. inconsistent)**.

We also found a replication of the moderated mediation model wherein caste norm condition was related to moral acceptability through the indirect effect of high-caste identity (*IE* = 0.11, 95% CI [0.01; 0.30]) while controlling for other identities. The results revealed that caste norm condition negatively predicted high-caste identity (*b* = -0.57, *t* = -2.53, *p* = 0.013, 95% CI [-1.02, -0.12]) and high caste identity negatively predicted moral acceptability (*b* = -0.19, *t* = -2.12, *p* = 0.039, 95% CI [-0.37, -0.01]). The conditional direct effect of caste norm condition on moral acceptability was again significant only among high-caste individuals (*b* = 1.84, *t* = 5.63, *p* < 0.0001, 95% CI [1.19, 2.48]) but not among low-caste individuals, (*b* = 0.13, *t* = 0.58, *p* = 0.561, 95% CI [-0.30, 0.56]). The interaction between condition and caste on status representation is graphically presented in **Figure 5**. The interaction between condition and caste is significant (*b* = 1.71, *t* = 4.17, *p* < 0.0001, 95% CI [0.90, 2.52]). Thus, we see that caste norm inconsistency leads to more high-caste identity which in turn results in lesser moral acceptability, however, only amongst high-caste individuals. The analyses were repeated for each of the other identities (religious, national, regional, and low caste) that were used as mediators while controlling for the other. The indirect effects for low-caste identity (*IE* = -0.04, 95% CI [-0.03; 0.11]), South Indian identity (*IE* = -0.01, 95% CI [-0.10; 0.04]), national identity (*IE* = 0.00, 95% CI [-0.03; 0.05]) and religious identity (*IE* = -0.00, 95% CI [-0.09; 0.01]) were again not significant.

**FIGURE 6:**
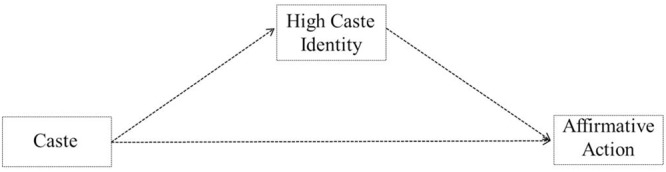
**Theoretical model of the relationship between caste (high vs. low), high caste identity and attitude toward affirmative action**.

To test the effects of actual caste on the social consequences of the caste system, such as attitudes toward affirmative action as mediated through upper caste identity, a mediation analysis was conducted (Process model 4, Hayes, 2013) as theoretically depicted in **Figure 6**. Again, in line with our predictions, the results showed that actual caste positively predicted high caste identity (*b* = 1.10, *t* = 4.78, *p* < 0.0001, 95% CI [0.65, 1.56]) and high caste identity negatively predicted the affirmative action attitudes (*b* = -0.39, *t* = -8.76, *p* < 0.0001, 95% CI [-0.53, -0.25]). We found an indirect effect of caste on affirmative action attitude partially mediated by high caste identity (*IE* = -0.43, 95% CI [0.07; 0.46]). The direct effect of actual caste on affirmative action attitudes was also significant (*b* = -1.56, *SE* = 0.18, *p* < 0.0001, 95% CI [-1.91, -1.21]). Thus, high caste leads to opposition of affirmative action partially due to heightened upper caste identity. The analyses were repeated for each of the other identities while controlling for the other. The indirect effects for lower caste identity (*IE* = 0.03, 95% CI [-0.20; 0.23]), South Indian identity (*IE* = -0.03, 95% CI [-0.06; 0.16]), national identity (*IE* = 0.02, 95% CI [-0.08; 0.15]) and religious identity (*IE* = -0.00, 95% CI [-0.08; 0.09]) were also not significant.

## Discussion

In this research, we apply a well-established theoretical framework incorporating social identity theory (Tajfel and Turner, 1986), the black sheep effect (Marques et al., 1988) and moral motives derived from relational models theory (Fiske, 1991; Rai and Fiske, 2011) to understand a small part of a complex social structure, namely the Indian caste system. It is important to state that we are not claiming that this study answered all the nuances seen in exploring the caste system but has paved the way toward a better understanding of this concept. Overall, we found support for our argument that group membership to a particular caste has consequences in the way one perceives status amongst fellow members in the society. More specifically, we validated that when high-caste individuals learn that their fellow in-group member engaged in norm-violating behavior then they devalue the status of that in-group member. Thus, for instance, when a high-caste member marries a low caste member, this high caste member will be devalued by his fellow in-groupers owing to norm-violation. Moreover, we find that this trend is particularly observed as a consequence of heightened high-caste identity. Our results show that in a situation of norm violation, high caste identity predicts the way people make status representations. We argue that this norm violation induces identity threat, especially among high-caste individuals and as a result of the threat they heighten their high-caste identity, thereby engaging in status devaluation. The stories in the study also depict an intentional aggressive, deviant behavior and in line with previous research (Abrams et al., 2000; Marques et al., 2001) this behavior led to black sheep effects in the form of status devaluation by high-caste members. Classic studies by Branscombe et al. (1993) and Biernat et al. (1999) show that highly identified individuals show more in-group derogation especially when one’s identity is threatened. Moreover, we also claim that since high-caste members tend to see caste identity as inherited at birth and also tend to essentialise their caste (Dube, 2001; Mahalingam, 2007) a high-caste identity will be more salient amongst the high-caste individuals than low caste-identity amongst the low-caste individuals. Thus, high caste members would especially defend the identity threat to maintain positive social identity due to heightened identification with the group (Marques and Paez, 1994) and would perceive the in-group deviant as less typical of the in-group (Castano et al., 2002).

Thus, in the present study we were able to show how status is represented without explicit information about the protagonist’s caste. The information people received was merely the person’s name and a photograph and yet it is evident that existing knowledge about caste was activated. As seen in the manipulation check, in general, most high-caste protagonist names were identified as belonging to the high caste and low-caste protagonist names as belonging to the low caste. Moreover, the study incorporated a novel method to measure perception of status by means of a projective task using the vertical dimension, as this dimension has been shown to be used in reasoning to simulate power (Schubert, 2005) and social status (von Hecker et al., 2013), in order to generate judgments on these characteristics. Therefore, when participants were depicting relations in such a way that a perpetrator was represented on the bottom of the vertical line and the victim on top, we believe the abstract concept of status and in-group derogation was clearly embodied.

It is important to understand that the reason a norm-violating social situation was chosen to induce identity threat was because in the Indian context caste norm violation is perhaps the most salient identity threat situation. Deshpande (2010) explains that caste has acquired hereditary characteristics in the Indian society which comes with certain norms that evolved from ancient scriptures. Thus, when these norms are violated in the society, it is received with punitive costs. The threat used in this context violated the basic morality values of the caste and according to Branscombe et al. (2000), threat to moral values leads to defensive reactions and perceived in-group heterogeneity, especially amongst high identifiers. Thus, when the high caste moral code of being virtuous and non-aggressive was violated, highly identified high caste members strengthened their identity and showed in-group derogation by devaluing the status of the in-group deviant. This reasoning was further confirmed when high-caste individuals in the norm-inconsistent (violating) condition showed lower moral acceptability of the behavior when the perpetrator was a high-caste member.

Secondly, the results also show that only high-caste individuals seem to be affected by the norm violation identity threat in making status representations but not the low-caste individuals. In fact, the low-caste individuals seem to show no differences in the way they represent status and moreover they seem to do so in a status-congruent direction, that is, the perpetrator is always depicted as having high status and a victim is always depicted as having low status. This could possibly be the case because historically low-caste individuals are the stigmatized low status groups (Mahalingam, 2003). Therefore, they probably do not experience the same amount of identity threat in a norm-violation condition as the high-caste individuals. The low caste individuals, due to the belief that their caste was not acquired through birth (Dube, 2001) and the lack of an established status, probably also showed lesser identification with their low-caste. Thus, when a low- caste individual reads a story about a norm-violating low-caste member who is in fact a victim, there is no threat that is activated probably because they do not show high identification. Furthermore, being portrayed as a victim does not threaten the image of the in-group of low caste individuals, thereby lowering the need to protect the in-group image.

Another important outcome of this study was that only high-caste identity seemed to play an integral role in predicting status effects and moral acceptability. As already explained by Judge and Bal (2008), caste identity dominates over all other identities in the Indian context, probably because of the inherent characteristics the concept of caste entails. As seen in the correlations (**Table 2**), only high caste and low caste identities seem to negatively correlate with each other; understandably because those with greater high caste identities would have lesser low caste identities. Interestingly, low-caste identity did not act as a significant mediator in predicting status relationships or understanding moral acceptability. Understandably because previous studies have shown that caste identity is essentialised and legitimized (Dube, 2001; Mahalingam, 2007) particularly by high caste individuals; thus possessing high-caste identity could result in understanding and representing status differences more than having low-caste identity. Our results also show that none of the other identities – national, regional or religious played an important role in predicting status distances or moral acceptability. While India is a complex social structure with an interactive role of multiple identities, we have reasonable evidence to believe that perhaps in the context of status representation, hierarchies and caste-related social outcomes, other identities are not as salient, so perhaps people discard these identities when being involved in status-relevant situations. Thus, it seems like in the context of how individuals see status in the society, only high-caste identity seems to be activated while alternative identities might be discarded. This is a very important finding because whilst studies have shown that caste identity is the most salient identity (Gayer, 2000), perhaps heightening other identities like regional or national identities could result in reducing caste-based status hierarchies or status representations. Interventions such as this could be seen in the work by Brewer and Gaertner (2008) where they speak of cross-categorization as a technique to overcome prejudice. A typical example could be seen in the sporting context when athletes all over the country from different socioeconomic backgrounds and castes come together as a team to play for the country. The heightened in-group national identity perhaps diminishes existing caste based identities that could potentially create situations of prejudice amongst various players. While there are many religious and regional conflicts we see that caste based discrimination is the most salient form when it involves differentiating groups of varied status.

Finally, the results also confirm the social consequences of a caste-based system in terms of affirmative action. As pointed out by Siddique (2011), affirmative action policies were introduced to improve the quality of life of low-caste individuals. However, it was soon met with opposition from the high-caste community. Sometimes caste is not always related to class, so it is possible that someone of low caste could be of high class and therefore have sufficient means and opportunities at places of education and government jobs. Thus, the present results confirmed that high-caste individuals would generally oppose affirmative action, whereby this link was partially mediated through high-caste identity. More importantly, again, none of the alternative identities played a role in determining attitudes toward affirmative action. This is unsurprising because affirmative action was introduced as an outcome of revisiting the consequence of the caste system for low-caste individuals. Therefore, other identities do not seem to play a vital role in predicting these effects.

The present study, however, has some limitations especially with respect to sample size and the sampling technique (convenience sampling). While only single-item measures were used for measuring identity, as a follow-up study caste-based identity should also be measured on its own using a questionnaire and relate this to the present findings. The present findings should also be extended to other norm-violating instances apart from the victim-perpetrator context. In India, several other norm violations exist, for instance, consuming beef in a purely ‘Hindu’ state, or women dressed in ‘western’ clothes, or engaging in inter-caste marriage, the latter being the most predominant caste-norm transgression. Furthermore, it would be essential to re-run the study, this time manipulating the class of the protagonists using different professions (without names), in order to rule out the influence of class in these processes. It would also be essential to replicate these findings with a North Indian sample. Finally, it is important to note that when a high caste individual is engaged in a norm-violation, there is a greater negative connotation as a high caste individual’s consistent norm is that of purity and morality (Deshpande, 2010). The low caste individuals’ consistent norm, however, is that of impurity and immorality, therefore, a negative expectation of their behavior is already established as a norm and therefore in a norm-violation condition, wherein they are portrayed as a victim, they are probably not as threatened as high-caste individuals. A positive connotation attached to a norm-violation perhaps does not induce as much threat and therefore low-caste individuals remain unaffected by the two conditions. This limitation is something that needs to be addressed in future studies.

## Conclusion

The present study tried to utilize basic social identity theories in explaining one outcome of a very complex social system, such as the Indian caste system. It also touched upon the most dominant identity that is useful in predicting status differences in the Indian society. Considering the complexity of the Indian caste system, it was crucial to look into a small consequence of the caste system rather than the whole structure on its own. This study also paves the way into the understanding of the black sheep effect as seen in status representation as supposed to general in-group derogation attitudes. It is fundamental to expand this study by examining other aspects of social identity threat in the Indian context, such as religion, to gain some more understanding of how multiple identities interact with each other in the functioning of the Indian society.

## Ethics Statement

The study was approved by the Scientific Research and Ethics Committee at the Institute of Psychology, Jagiellonian University, Krakow, Poland (the date of the record July 2, 2014). All participants signed the informed consent form and were told that they were free to withdraw from the study at any time.

## Author Contributions

SS and UvH initially conceptualized the idea. SS and MS developed the idea and primarily designed the study. SS was mainly involved in data collection and data analyses. SS and MS were involved in data interpretation and UvH contributed to the discussion. SS was involved in writing the paper draft of the paper. Both MS and UvH were involved in the revised versions of paper.

## Conflict of Interest Statement

The authors declare that the research was conducted in the absence of any commercial or financial relationships that could be construed as a potential conflict of interest.

## Footnotes

1 Caste is not always related to socioeconomic class even though the two constructs are connected. For instance, a high caste and a high class individual could be a university professor while a high caste and a low class individual could be a *Brahmin* temple priest. Similarly, a politician could be a low caste and a high class individual and a typical example of a low caste, low class individual would be household help or a flower seller.

2 The terms *backward* and *forward* are used based on the official classification issued by the government of India and recognized in the Constitution of India. Essentially the backward caste groups comprise of historically disadvantaged people in India while the forward caste comprise those of the more privileged high caste individuals. These terms by no means denote in the linguistic sense of moving forward or backward.

3 We would like to argue that participants in fact have a pre-disposed idea of status based on information about caste. Thus, while the stories in the vignettes do imply a power relation, this pre-disposed notion of status has short term influence in making status representations. We argue that the transgression as an outcome of power relation is still evaluated based on which person is perpetrator –the high or low caste individual.

4 Apart from the two South Indian stories, all participants also had to respond to two other stories that were pertaining to the North Indian context. Since the participants were all from Chennai, Tamil Nadu in South India we did not include the distance and moral acceptability scores of the North Indian stories. Furthermore, the manipulation check reveled that when asked to classify the protagonists as belonging to a particular caste, most North Indian protagonists were classified in the ‘unsure’ category. Thus we only used the data pertaining to the South Indian context.

## References

[B1] Abrams D. Marques J. M. Bown N. Henson M. (2000). Pro-norm and anti-norm deviance within and between groups. *J. Pers. Soc. Psychol.* 78 906–912. 10.1037/0022-3514.78.5.906 10821197

[B2] Ambedkar B. R. (1925/1989). “Essays on untouchables and untouchability I,” In *Writings and Speeches* Vol. 5 ed. Moon V. (Bombay: Education Department, Government of Maharashtra).

[B3] Biernat M. Vescio T. K. Billings L. S. (1999). Black sheep and expectancy violation: integrating two models of social judgment. *Eur. J. Soc. Psychol.* 29 523–542. 10.1002/(SICI)1099-0992(199906)29:4<523::AID-EJSP944>3.0.CO;2-J

[B4] Branscombe N. A. Castle K. Dorsey A. G. Surbeck E. Taylor J. B. (2000). *Early Childhood Education – A Constructivist Approach.* Boston: Houghton Mifflin Company.

[B5] Branscombe N. R. Ellemers N. Spears R. Doosje B. (1999). “The context and content of social identity threat,” in *Social Identity: Context, Commitment, Content* eds Ellemers N. Spears R. Doosje B. (Oxford, UK: Blackwell) 35–58.

[B6] Branscombe N. R. Wann D. L. Noel J. G. Coleman J. (1993). In-Group or out-group extremity: importance of the threatened social identity. *Pers. Soc. Psychol. Bull.* 19 381–388. 10.1177/0146167293194003

[B7] Brewer M. B. Gaertner S. L. (2008). “Toward reduction of prejudice: intergroup contact and social categorization,” in *Blackwell Handbook of Social Psychology: Intergroup Processes* eds Brown R. Gaertner S. L. (Oxford: Blackwell Publishers Ltd) 10.1002/9780470693421.ch22

[B8] Castano E. Paladino M.-P. Coull A. Yzerbyt V. Y. (2002). Protecting the ingroup stereotype: ingroup identification and the management of deviant ingroup members. *Br. J. Soc. Psychol.* 41 365–385. 10.1348/014466602760344269 12419008

[B9] Cotterill S. Sidanius J. Bhardwaj A. Kumar V. (2014). Ideological support for the indian caste system: social dominance orientation, right-wing authoritarianism and karma. *J. Soc. Polit. Psychol.* 2 98–116. 10.5964/jspp.v2i1.171

[B10] Deshpande M. S. (2010). *History of the Indian Caste System and Its Impact on India Today.* New York, NY: California University Press.

[B11] Dirks N. B. (1989). The original caste: power, history and hierarchy in south asia. *Contrib. Indian Sociol.* 23 59–77. 10.1177/006996689023001005

[B12] Doosje B. Ellemers N. Spears R. (1995). Perceived intragroup variability as a function of group status and identification. *J. Exp. Soc. Psychol.* 31 410–436.

[B13] Dreze J. Khera R. (2009). *The Battle for Employment Guarantee.* Available at: http://www.frontline.in/static/html/fl2601/stories/20090116260100400.htm

[B14] Dube L. (2001). *Anthropological Explorations in Gender: Intersecting Fields.* Thousand Oaks, CA: SAGE.

[B15] Duncan B. L. (1976). Differential social perception and attribution of intergroup violence: testing the lower limits of stereotyping of Blacks. *J. Pers. Soc. Psychol.* 34 590–598. 10.1037/0022-3514.34.4.590 993976

[B16] Fiske A. P. (1991). *Structures of Social Life: The Four Elementary Forms of Human Relations.* New York, NY: Free Press.

[B17] Fiske A. P. (1992). The four elementary forms of sociality: framework for a unified theory of social relations. *Psychol. Rev.* 99 689–723. 10.1037/0033-295x.99.4.689 1454904

[B18] Flintoff C. (2010). *India Struggles to Stem Rise in Honor Killings.* Available at: http://www.npr.org/templates/story/story.php?storyId=128567642

[B19] Gayer L. (2000). The globalization of identity politics: the Sikh experience. *Int. J. Punjab Stud.* 7 223–262.

[B20] Giessner S. R. Schubert T. W. (2007). High in the hierarchy: how vertical location and judgments of leaders’ power are interrelated. *Organ. Behav. Hum. Decis. Process.* 104 30–44. 10.1016/j.obhdp.2006.10.001

[B21] Goli S. Singh D. Sekher T. (2013). Exploring the myth of mixed marriages in India: evidence from a nation-wide survey. *J. Comp. Fam. Stud.* 44 193–206.

[B22] Gupta D. (2005). Caste and politics: identity over system. *Annu. Rev. Anthropol.* 34 409–427. 10.1146/annurev.anthro.34.081804.120649

[B23] Hayes A. F. (2013). *Introduction to Mediation, Moderation, and Conditional Process Analysis: A Regression-Based Approach.* New York, NY: The Guilford Press.

[B24] Hoff K. Kshetramade M. Fehr E. (2009). *Caste and Punishment: The Legacy of Caste Culture in Norm Enforcement. Policy Research Working Papers.* Washington, DC: World Bank 10.1596/1813-9450-5040

[B25] Hogg M. A. Turner J. C. (1987). Intergroup behavior, self-stereotyping and the salience of social categories. *Br. J. Soc. Psychol.* 26 325–340. 10.1111/j.2044-8309.1987.tb00795.x

[B26] Jaspal R. (2011). Caste, social stigma and identity processes. *Psychol. Dev. Soc.* 23 27–62. 10.1177/097133361002300102

[B27] Jetten J. Hornsey M. J. (2014). Deviance and dissent in groups. *Annu. Rev. Psychol.* 65 461–485. 10.1146/annurev-psych-010213-115151 23751035

[B28] Judge P. S. Bal G. (2008). Understanding the paradox of changes among Dalits in Punjab. *Econ. Polit. Wkly.* 43 49–55.

[B29] Kumar V. (2001). Untouchability in Uttaranchal. *Econ. Polit. Wkly.* 36 4536–4537.

[B30] Mahalingam R. (2003). Essentialism, culture, and power: rethinking social class. *J. Soc. Issues* 59 733–749. 10.1046/j.0022-4537.2003.00087.x

[B31] Mahalingam R. (2007). Beliefs about chastity, machismo, and caste identity: a cultural psychology of gender. *Sex Roles* 56 239–249. 10.1007/s11199-006-9168-y

[B32] Mand K. (2006). Gender, ethnicity and social relations in the narratives of elderly Sikh men and women. *Ethn. Racial Stud.* 29 1057–1071. 10.1080/01419870600960305

[B33] Marques J. Abrams D. Paez D. Martinez-Taboada C. (1998). The role of categorization and in-group norms in judgments of groups and their members. *J. Pers. Soc. Psychol.* 75 976–988. 10.1037/0022-3514.75.4.976

[B34] Marques J. Abrams D. Serôdio R. G. (2001). Being better by being right: subjective group dynamics and derogation of in-group deviants when generic norms are undermined. *J. Pers. Soc. Psychol.* 81 436–447. 10.1037/0022-3514.81.3.436 11554645

[B35] Marques J. Yzerbyt V. Leyens J. P. (1988). The ‘black sheep effect’: extremity of judgments toward ingroup members as a function of group identification. *Eur. J. Soc. Psychol.* 18 1–16. 10.1002/ejsp.2420180102

[B36] Marques J. M. Paez D. (1994). “The black sheep effect: social categorisation, rejection of ingroup deviates, and perception of group variability,” in *European Review of Social Psychology* Vol. 5 eds Stroebe W. Hewstone M. (New York, NY: Wiley) 38–68.

[B37] Miller J. G. Bersoff D. M. (1992). Culture and moral judgment: how are conflicts between justice and interpersonal responsibilities resolved? *J. Pers. Soc. Psychol.* 62 541–554. 10.1037/0022-3514.62.4.541 1583583

[B38] Miller J. G. Bersoff D. M. Harwood R. L. (1990). Perceptions of social responsibilities in India and in the United States: moral imperatives or personal decisions? *J. Pers. Soc. Psychol.* 58 33–47. 10.1037/0022-3514.58.1.33 2308074

[B39] Niedenthal P. M. Barsalou L. W. Winkielman P. Krauth-Gruber S. Ric F. (2005). Embodiment in Attitudes. *Soc. Percept. Emot. Pers. Soc. Psychol. Rev.* 9 184–211. 10.1207/s15327957pspr0903_1 16083360

[B40] Otten S. (2009). “Social categorization, intergroup emotions and aggressive interactions,” in *Intergroup Relations: The Role of Motivation and Emotion* eds Otten S. Sassenberg K. Kessler T. (New York, NY: Psychology Press) 162–181.

[B41] Pick D. Dayaram K. (2006). Modernity and tradition in a global era: the re-invention of caste in India. *Int. J. Sociol. Soc. Policy* 26 284–294. 10.1108/01443330610680380

[B42] Pinto I. R. Marques J. M. Levine J. M. Abrams D. (2010). Membership status and subjective group dynamics: who triggers the black sheep effect? *J. Pers. Soc. Psychol.* 99 107–119. 10.1037/a0018187 20565188

[B43] Pratto F. Liu J. H. Levin S. Sidanius J. Shih M. Bachrach H. (2000). Social dominance orientation and the legitimization of inequality across cultures. *J. Cross Cult. Psychol.* 31 369–409. 10.1177/0022022100031003005

[B44] Rai T. S. Fiske A. P. (2011). Moral psychology is relationship regulation: moral motives for unity, hierarchy, equality, and proportionality. *Psychol. Rev.* 118 57–75. 10.1037/a0021867 21244187

[B45] Rimal R. N. Real K. (2005). How behaviors are influenced by perceived norms. *Commun. Res.* 32 389–414. 10.1177/0093650205275385

[B46] Sagar A. Schofield J. W. (1980). Racial and behavioral cues in Black and White children’s perceptions of ambiguously aggressive acts. *J. Pers. Soc. Psychol.* 39 590–598. 10.1037/0022-3514.39.4.590 7431207

[B47] Schubert T. W. (2005). Your highness: vertical positions as perceptual symbols of power. *J. Pers. Soc. Psychol.* 89 1–21. 10.1037/0022-3514.89.1.1 16060739

[B48] Sheth D. L. (1987). Reservations policy revisited. *Econ. Polit. Wkly.* 22 461957–461962.

[B49] Sidanius J. Pratto F. (1999). *Social Dominance: An Intergroup Theory of Social Hierarchy and Oppression.* New York, NY: Cambridge University Press 10.1017/CBO9781139175043

[B50] Siddique Z. (2011). Evidence on caste based discrimination. *Labour Econ.* 18 S146–S159. 10.1016/j.labeco.2011.07.002

[B51] Spears R. Doosje B. Ellemers N. (1997). Self-stereotyping in the face of threats to group status and distinctiveness: the role of group identification. *Pers. Soc. Psychol. Bull.* 23 538–553. 10.1177/0146167297235009

[B52] Stamkou E. van Kleef G. A. Homan A. C. Galinsky A. D. (2016). How norm violations shape social hierarchies: those who stand on top block norm violators from rising up. *Group Process. Intergroup Relat.* 19 608–629. 10.1177/1368430216641305

[B53] Tajfel H. Turner J. C. (1986). “The social identity theory of intergroup behavior,” in *The Social Psychology of Intergroup Relations* eds Worchel S. Austin W. (Chicago, IL: Nelson–Hall) 7–24.

[B54] von Hecker U. Klauer K. C. Sankaran S. (2013). Embodiment of social status: verticality effects in multilevel rank-orders. *Soc. Cogn.* 31 374–389. 10.1521/soco_2013_1006

[B55] Wang L. Zheng J. Meng L. Lu Q. Ma Q. (2016). Ingroup favoritism or the black sheep effect: perceived intentions modulate subjective responses to aggressive interactions. *Neurosci. Res.* 108 46–54. 10.1016/j.neures.2016.01.011 26851770

[B56] “What is India’s caste system?,” (2016). Available at: http://www.bbc.com/news/world-asia-india-35650616

