# Supplementary material for: The Role of Indian Caste Identity and Caste Inconsistent Norms on Status Representation
Source: Front Psychol. 2017 Mar 31;8:487. doi: 10.3389/fpsyg.2017.00487 (PMC5374864; doi:10.3389/fpsyg.2017.00487)
Supplement: Supplementary file 1 [file Data_Sheet_1.docx]

**Appendix 1**

*Traffic Quarrel-Norm Consistent*

At the junction of Mount Road there was a huge traffic jam where two cars collided. While nobody got hurt, the drivers jumped out of their respective cars and started hurling verbal abuses at each other. That quarrel soon turned into a proper fight when Selvaraj Mani jumped out from his car and attacked Murali Raman. Murali Raman was pulled out of his car and was punched hard by Selvaraj Mani. Murali Raman finally fell to the ground and shouted apologies, but Selvaraj Mani did not stop kicking him until the police came and pulled the two drivers apart.

*Traffic Quarrel-Norm Inconsistent*

At the junction of Mount Road there was a huge traffic jam where two cars collided. While nobody got hurt, the drivers jumped out of their respective cars and started hurling verbal abuses at each other. That quarrel soon turned into a proper fight when Murali Raman jumped out from his car and attacked Selvaraj Mani. Selvaraj Mani was pulled out of his car and was punched hard by Murali Raman. Selvaraj Mani finally fell to the ground and shouted apologies, but Murali Raman did not stop kicking him until the police came and pulled the two drivers apart.

*Hostile Work Situation-Norm Consistent*

Kannan Pandian and Ravi Krishnamurthy work on the same floor in a branch of a large international corporation. One they both were in a hurry for an important meeting and needed to use the photocopier machine. Unfortunately, the copier was out of order. They both got impatient and upset and Kannan Pandian immediately started accusing Ravi Krishnamurthy of breaking the machine. Ravi Krishnamurthy denied it and tried explaining calmly to Kannan Pandian that the photocopier was broken before either of them got to use it. However, Kannan Pandian was very angry and would not listen but instead turned his back on Ravi Krishnamurthy and went away muttering insults.

*Hostile Work Situation-Norm Inconsistent*

Ravi Krishnamurthy and Kannan Pandian work on the same floor in a branch of a large international corporation. One they both were in a hurry for an important meeting and needed to use the photocopier machine. Unfortunately, the copier was out of order. They both got impatient and upset and Ravi Krishnamurthy immediately started accusing Kannan Pandian of breaking the machine. Kannan Pandian denied it and tried explaining calmly to Ravi Krishnamurthy that the photocopier was broken before either of them got to use it. However, Ravi Krishnamurthy was very angry and would not listen but instead turned his back on Kannan Pandian and went away muttering insults.
